# Acute phase responses induced in dwarf goats by r.BolL_−1β_, r.BolL_−2_ and r.BolFN_−γ_

**DOI:** 10.1155/S0962935192000310

**Published:** 1992

**Authors:** C. T. M. van Duin, T. Wensing, A. S. J. P. A. M. van Miert

**Affiliations:** 1 Department of Veterinary Basic Sciences Division of Pharmacology Pharmacy and Toxicology Faculty of Veterinary Medicine, Utrecht University, P.O. Box 80176 Utrecht 3508 TD The Netherlands; 2 Department of Large Animal Medicine Faculty of Veterinary Medicine Utrecht University P.O. Box 80176 Utrecht The Netherlands

## Abstract

The contribution of each of the pro-inflammatory cytokines to specific components of the host response to infection remains unclear. Therefore, the effects of single doses of cytokines were studied in dwarf goats. The present study was carried out to investigate the effects of r.BoIL_−1β_, r.BoIL_−2_ and r.BoIFN_−γ_ on plasma zinc and iron concentrations, white blood cell counts, and body temperature. The i.v. injection of r.BolL_−1β_ (1 μg kg^−1^) resulted in an immediate fever which reached peak values 45 and 180 min after injection. Compared with fever induced by r.BoIL_−1β_, that caused by r.BoIFN_−γ_ (2 μg kg^−1^) was delayed in onset. Although the biphasic fever after r.BoIFN_−γ_ was more pronounced than after r.BoIL_−1β_, the reduction in plasma trace metal concentrations was less than after r.BoIL_−1β_, r.BoIL_−2_ (1 μg kg^−1^ i.v.) did not induce changes in these parameters. The haematologic changes observed revealed a cell type and cytokine specific pattern. The delayed onset of the effects induced by IFN_−γ_ suggests that they may be mediated through the induction of other mediators of inflammation.


Mediators of Inflammation, 1, 201–206 (1992)

### Acute phase responses induced in dwarf goats by $r.BoIL_{-1\beta}$, $r.BoIL_{-2}$ and $r.BoIFN_{-\gamma}$

C. T. M. van Duin,[1] T. Wensing[2] and A. S. J. P. A. M. van Miert[1,CA]

[1]Department of Veterinary Basic Sciences, Division of Pharmacology, Pharmacy and Toxicology, and [2]Department of Large Animal Medicine, Faculty of Veterinary Medicine, Utrecht University, P.O. Box 80176, 3508 TD Utrecht, The Netherlands

[CA] Corresponding Author

**THE contribution of each of the pro-inflammatory cytokines to specific components of the host response to infection remains unclear. Therefore, the effects of single doses of cytokines were studied in dwarf goats. The present study was carried out to investigate the effects of $r.BoIL_{-1\beta}$, $r.BoIL_{-2}$ and $r.BoIFN_{-\gamma}$ on plasma zinc and iron concentrations, white blood cell counts, and body temperature. The i.v. injection of $r.BoIL_{-1\beta}$ (1 $\mu g \cdot kg^{-1}$) resulted in an immediate fever which reached peak values 45 and 180 min after injection. Compared with fever induced by $r.BoIL_{-1\beta}$, that caused by $r.BoIFN_{-\gamma}$ (2 $\mu g \cdot kg^{-1}$) was delayed in onset. Although the biphasic fever after $r.BoIFN_{-\gamma}$ was more pronounced than after $r.BoIL_{-1\beta}$, the reduction in plasma trace metal concentrations was less than after $r.BoIL_{-1\beta}$. $r.BoIL_{-2}$ (1 $\mu g \cdot kg^{-1}$ i.v.) did not induce changes in these parameters. The haematologic changes observed revealed a cell type and cytokine specific pattern. The delayed onset of the effects induced by $IFN_{-\gamma}$ suggests that they may be mediated through the induction of other mediators of inflammation.**

**Key words:** Acute phase response, Dwarf goat, Fever, Hypoferraemia, Hypozincaemia, $r.BoIL_{-1\beta}$, $r.BoIL_{-2}$, $r.BoIFN_{-\gamma}$, WBC

### Introduction

In mammals, tissue damage, inflammation or invasion by pathogenic micro-organisms induces systemic changes, collectively known as the acute phase response. Among the varied alterations, which together produce this response, are fever, decreased plasma iron and zinc concentrations and changes in white blood cell counts.[1–4] Bacteria require large amounts of iron and zinc for cell growth, particularly at elevated temperature, and the ability of the host to remove these trace elements from tissue fluids seems to be a fundamental host defence mechanism.[4–6] The intensity of the body temperature response, the decrease in trace metal concentrations and the change in white blood cell counts (WBC) may vary depending on the type of invading micro-organism or bacterial toxin given.[3,4] Such host responses to infection or injury are thought to be caused by the endogenous synthesis and release of a variety of cytokines, including tumour necrosis $factor_{-\alpha}$ ($TNF_{-\alpha}$), interleukins ($IL_{-1}$, $IL_{-6}$), and interferons ($IFN_{-\alpha}$, $IFN_{-\gamma}$). These cytokines have been detected in serum[7–11] and cerebrospinal fluid[12–14] in several diseases. Moreover, they have been found to be pyrogenic.[2,15] Furthermore, cancer patients undergoing treatment with high dose recombinant $IL_{-2}$ develop serious side effects including profuse sweats and fever.[16,17] *In vivo*, high doses of $r.IL_{-2}$ induce TNF in cancer patients;[18] *in vitro*, $r.IL_{-2}$ induces both TNF and $IFN_{-\gamma}$ from human mononuclear cells.[19] The contribution of each of these individual cytokines to specific components of the host response to infection, inflammation and endotoxaemia remains unclear. We examined, therefore, the effects of single doses of cytokines on body temperature, food intake, gastric function, heart rate, plasma zinc and iron concentrations, and on white blood cell counts. In previous studies, the effects of dwarf goats to *Escherichia coli* endotoxins, $r.HuIFN_{-\alpha 2a}$, $r.BoTNF_{-\alpha}$ and $r.HuTNF_{-\alpha}$ have been reported.[20–25] In this study, we present the effects of $r.BoIL_{-1\beta}$, $r.BoIL_{-2}$ and $r.BoIFN_{-\gamma}$ on body temperature, WBC and plasma Fe and Zn levels. The data on heart rate, gastric function and food intake will be reported elsewhere.[23]

### Materials and Methods

*Animals:* For the present studies, eighteen healthy dwarf goats—females and castrated males—were used. The animals were trained to stand quietly during recording sessions by repeatedly placing them in conventional goat restraint boxes for several hours daily. Thereafter, the goats were allocated to three groups; group I goats ($n = 6$) weighed between 20 and 35.5 kg (mean ± SE: 27.2 ± 2.25 kg), group II goats ($n = 6$) weighed between 20.5 and 34.5 kg (mean ± SE: 26.90 ± 2.03 kg), and group III goats ($n = 6$) weighed

© 1992 Rapid Communications of Oxford Ltd between 41.4 and 45 kg (mean $\pm$ SE: 43.3 $\pm$ 0.59 kg). Group I goats were treated i.v. with r.BoIL$_{-1\beta}$ (1 $\mu$g·kg$^{-1}$ or 3.45 $\times$ $10^4$ IU kg$^{-1}$), group II goats were treated i.v. with r.BoIL$_{-2}$ (1 $\mu$g·kg$^{-1}$ or 2.28 $\times$ $10^4$ IU kg$^{-1}$), and r.BoIFN$_{-\gamma}$ was given as an i.v. infusion over 30 min (total dose 2 $\mu$g·kg$^{-1}$ or 6.0 $\times$ $10^3$ U kg$^{-1}$) into group III goats. All animals were kept indoors and fed a diet of hay and pelleted concentrate. Water was provided freely.

*Rectal temperature measurements:* Rectal temperature of the goats was measured with a thermistor-based electronic thermometer (Scanning tele-thermometer, model 47, Yellow Springs Instrument Corp. Inc., Yellow Springs, OH, USA); the appropriate thermistors (probe no. 401) were inserted at least 8 cm into the rectum. Temperatures were read at 15 min intervals for a minimum of 6 h.

*Blood analyses:* WBC and plasma zinc and iron concentrations were determined by methods described previously.[24,25] Samples of blood were collected in vacutainer tubes (Venoject®, Terumo Corp., Tokyo, Japan) by puncture of the jugular vein. The blood from these samples was heparinized (143 USP units sodium heparin per tube) and, after centrifugation, the plasma was stored at $-20$°C until analysed for zinc and iron concentrations. Blood samples (5 ml each) were obtained before, and at 1, 3.5, 8, 12 and 24 h after the cytokines were given. Results were expressed in percentages of the pre-injection values.

*Recombinant bovine cytokines:* Although two IL$_{-1}$ species, IL$_{-1\alpha}$ and IL$_{-1\beta}$, have been identified, they act on similar target cells via common receptors. IL$_{-1\beta}$ is the principal secreted form, whereas IL$_{-1\alpha}$ remains cell associated. Both IL$_{-1\alpha}$ and IL$_{-1\beta}$ bind to IL$_{-1}$ receptors with equal affinity and induce similar biological responses when injected *in vivo.*[19] In the present study, r.BoIL$_{-1\beta}$ was used. The r.BoIL$_{-1\beta}$ and r.BoIL$_{-2}$ were obtained from American Cyanamid Company, Princeton, NJ, USA (Immunex, Seattle, WA, USA). These stock solutions (lot no. 7818 c-166 W and 051289, respectively) contained 6.8 mg protein per ml with a titre of 3.45 $\times$ $10^7$ IU per mg protein, and 3.945 mg protein per ml with a titre of 2.28 $\times$ $10^7$ IU per mg protein, respectively. The r.BoIFN$_{-\gamma}$ was synthesized in *E. coli* by recombinant DNA technology and purified to homogeneity as determined by high-pressure liquid chromatography and sodium dodecylsulphate—polyacrylamide gel electrophoresis (Ciba-Geigy Ltd, Basel, Switzerland). The stock solutions (code CGA 212244, lot no. 3-2328) contained 5 mg protein per ml with a titre of 3.0 $\times$ $10^6$ U per mg protein when titrated on MDBK cells challenged with vesicular stomatitis virus and calibrated to laboratory reference standards. To obtain adequate information about the biological effects of r.BoIL$_{-1\beta}$, r.BoIL$_{-2}$ and r.BoIFN$_{-\gamma}$, we used doses similar to those reported to be effective in rabbits[18,26] and cattle.[27] Due to a limited number of goats, we did not perform dose—effect titration studies, as r.Bo cytokines may induce specific antibodies when repeatedly used in goats.

*Statistical analysis:* Significance of differences was tested with Student's paired *t*-test or independent *t*-test, where appropriate.

### Results

After i.v. administration, r.BoIL$_{-1\beta}$ and r.BoIFN$_{-\gamma}$ caused shivering, occurring as two different episodes, and biphasic temperature responses. As shown in Figure 1, a bolus injection of 1 $\mu$g·kg$^{-1}$ r.BoIL$_{-1\beta}$ induced immediate fever which reached peak values 45 min and 180 min after the injection. Thereafter, the temperature responses gradually returned to normal values. Shivering accompanied the onset of fever occurring after 3 to 9 min (mean $\pm$ SEM: 6 $\pm$ 0.8 min). In contrast, r.BoIFN$_{-\gamma}$ (2 $\mu$g·kg$^{-1}$) induced shivering after 18 to 40 min (mean $\pm$ SEM: 26 $\pm$ 3.5 min). Although the temperature responses were biphasic, peaks occurred much later than after r.BoIL$_{-1\beta}$ at 90 min and 315 min after r.BoIFN$_{-\gamma}$ administration (Figure 1). Injections of r.BoIL$_{-2}$ at a dose of 1 $\mu$g·kg$^{-1}$ were nonpyrogenic in dwarf goats.

Within 4 h of giving r.BoIL$_{-1\beta}$ to the goats, significant decreases were seen in plasma zinc and iron concentrations (mean $\pm$ SEM in per cent from baseline values: 65 $\pm$ 4 and 82 $\pm$ 4 at 3.5 h, respectively; Figure 1), with lowest values occurring after 8 h (Zn: 34 $\pm$ 3%) and 12 h (Fe: 33 $\pm$ 3%), respectively. The decrease in plasma concentrations of these trace metals was less pronounced in goats given r.BoIFN$_{-\gamma}$, with lowest values occurring after 12 h (Zn: 61 $\pm$ 3%, Fe: 72 $\pm$ 8%). Thus, the reduction in both plasma Zn and Fe concentrations was less clear ($p < 0.05$) and less persistent after r.BoIFN$_{-\gamma}$ injection than after r.BoIL$_{-1\beta}$ administration; at 24 h, in per cent from baseline values, plasma Zn and Fe values were: 93 $\pm$ 4 and 76 $\pm$ 3 after r.BoIFN$_{-\gamma}$, and 67 $\pm$ 5 and 43 $\pm$ 8 after r.BoIL$_{-1\beta}$, respectively; $p < 0.05$. In contrast, the febrile reactions after i.v. r.BoIFN$_{-\gamma}$ were more pronounced ($p < 0.05$; Figure 1) than after r.BoIL$_{-1\beta}$ which indicates that, in goats, IFN$_{-\gamma}$ is a potent pyrogenic cytokine. These results suggest that there is no causal relationship between body temperature responses and the alterations in trace metal concentrations in plasma. No significant changes in plasma Zn and Fe values were found after i.v. r.BoIL$_{-2}$ administration (Figure 1).

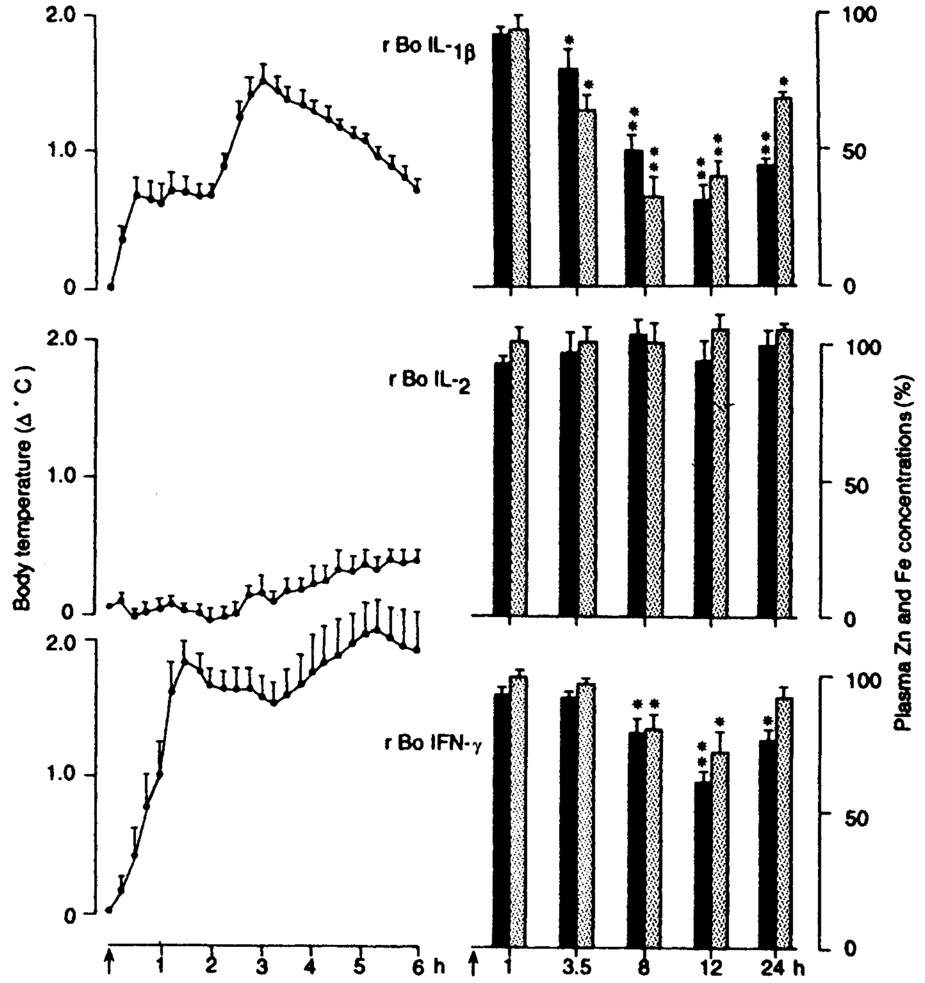

FIG. 1. Mean changes ($\pm$SEM) in rectal temperature and plasma iron (black bars) and zinc (dotted bars) concentrations after i.v. administration of r.BoIL$_{-1\beta}$ (1 μg·kg$^{-1}$; $n = 6$), r.BoIL$_{-2}$ (1 μg·kg$^{-1}$; $n = 6$) or r.BoIFN$_{-\gamma}$ (2 μg·kg$^{-1}$; $n = 6$). The mean rectal temperature in the three groups before they were treated was 38.55 $\pm$ 0.10°C, 38.49 $\pm$ 0.05°C and 38.48 $\pm$ 0.09°C, respectively. The baseline plasma Zn and Fe concentrations in the IL$_{-1\beta}$ group were 9.2 $\pm$ 0.83 μmol·l$^{-1}$ and 20.7 $\pm$ 2.42 μmol·l$^{-1}$, respectively. In the IL$_{-2}$ group, these pretreatment values were 8.5 $\pm$ 0.26 μmol·l$^{-1}$ and 19.3 $\pm$ 1.10 μmol·l$^{-1}$; in the IFN$_{-\gamma}$ group: 8.7 $\pm$ 0.26 μmol·l$^{-1}$ and 22.9 $\pm$ 1.02 μmol·l$^{-1}$. *$p < 0.05$, **$p < 0.01$: Significant difference from baseline values.

Table 1 summarizes the changes in the number of the circulating white blood cells after i.v. injection of the cytokines tested. Only r.BoIL$_{-1\beta}$ caused a short-lasting leukopenia, which was followed by a significant and long-lasting increase in the number of circulating leukocytes. Between 8 and 12 h after treatment with r.BoIL$_{-2}$ or r.BoIFN$_{-\gamma}$, leukocyte counts increased above pretreatment values. Lymphocyte counts decreased after administration of all cytokines tested with lowest values occurring at 3.5 h after r.BoIL$_{-1\beta}$ or r.BoIL$_{-2}$ administration. r.BoIFN$_{-\gamma}$ lowered lymphocyte counts to a mean ($\pm$ SEM) of 53 $\pm$ 8.1% of pretreatment values at 8 h. Only r.BoIL$_{-2}$ caused a short-lasting lymphopenia; lymphocyte counts returned to pretreatment values within 24 h after exposure to r.BoIL$_{-1\beta}$ and r.BoIFN$_{-\gamma}$.

Analysis of neutrophil counts and band forms before and after injection of cytokines revealed a different pattern (Table 1). The most prominent changes were observed after treatment with r.BoIL$_{-1\beta}$. The short-lasting neutropenia at 1 h was followed by a marked and long-lasting increase in neutrophil counts. Treatment with all cytokines led to an increase of neutrophil counts peaking at 12 h after injection. Increments after administration of r.BoIL$_{-2}$ and r.BoIFN$_{-\gamma}$ were not accompanied by an increase in the numbers of band forms in the peripheral blood. In contrast, r.BoIL$_{-1\beta}$ induced a significant increment of band forms with highest values occurring between 8 and 12 h (absolute counts $\times 10^3$ per μl at 8 h; 0.89 $\pm$ 0.11; at 12 h, 0.97 $\pm$ 0.35; and before cytokine injection, 0.05 $\pm$ 0.03). Thus, the haematologic changes

**Table 1.** Counts of total white blood cells (WBC), lymphocytes and neutrophils in goats (in each group $n = 6$) treated intravenously with r.BoIL-$_{1\beta}$ (1 $\mu g \cdot kg^{-1}$ b.w.), r.BoIL-$_2$ (1 $\mu g \cdot kg^{-1}$ b.w.) or r.BoIFN-$_\gamma$ (2 $\mu g \cdot kg^{-1}$ b.w.)

| | Per cent from baseline values | | | | | |
|---|---|---|---|---|---|---|
| | 0 h (Baseline) | 1 h | 3.5 h | 8 h | 12 h | 24 h |
| **WBC** | | | | | | |
| (Baseline × $10^3$ per $\mu l$) | | | | | | |
| r.BoIL-$_{1\beta}$ | 8.52 ± 0.92 | 56 ± 4.3* | 95 ± 7.3 | 171 ± 17.1* | 191 ± 20.8* | 178 ± 15.0* |
| r.BoIL-$_2$ | 7.87 ± 0.79 | 95 ± 3.6 | 106 ± 11.9 | 128 ± 8.1* | 129 ± 9.0* | 100 ± 9.1 |
| r.BoIFN-$_\gamma$ | 7.83 ± 0.71 | 85 ± 5.3* | 91 ± 7.8 | 122 ± 10.7 | 138 ± 14.0* | 129 ± 13.6 |
| **Lymphocytes** | | | | | | |
| (Baseline × $10^3$ per $\mu l$) | | | | | | |
| r.BoIL-$_{1\beta}$ | 4.43 ± 0.73 | 72 ± 9.1* | 50 ± 9.2* | 71 ± 7.2* | 74 ± 10.1* | 98 ± 18.8 |
| r.BoIL-$_2$ | 4.40 ± 0.51 | 89 ± 4.9 | 81 ± 5.9* | 121 ± 13.7 | 98 ± 10.3 | 100 ± 12.9 |
| r.BoIFN-$_\gamma$ | 4.15 ± 0.30 | 75 ± 4.5* | 60 ± 7.3* | 53 ± 8.1* | 68 ± 9.4* | 95 ± 6.7 |
| **Neutrophils** | | | | | | |
| (Baseline × $10^3$ per $\mu l$) | | | | | | |
| r.BoIL-$_{1\beta}$ | 3.83 ± 0.25 | 44 ± 5.7* | 144 ± 14.3* | 242 ± 43.3* | 301 ± 49.8* | 259 ± 35.1* |
| r.BoIL-$_2$ | 3.22 ± 0.78 | 107 ± 12.1 | 186 ± 56.5 | 149 ± 31.8 | 190 ± 23.1* | 103 ± 13.9 |
| r.BoIFN-$_\gamma$ | 3.46 ± 0.44 | 101 ± 8.3 | 133 ± 14.7 | 209 ± 19.2* | 221 ± 27.8* | 171 ± 25.4* |

Data are expressed as mean ± SEM; *$p < 0.05$.

observed in dwarf goats after r.BoIL-$_{1\beta}$, r.BoIL-$_2$ and r.BoIFN-$_\gamma$ revealed a cell type and cytokine specific pattern.

### Discussion

The acute phase response to bacterial infection is associated with the production of a complex cascade of cytokines that direct metabolic and immunological responses in the host.[1,28,29] Attention has been focused on defining the precise biological functions of individual cytokines, with TNF-$_\alpha$, IL-$_1$ and IL-$_6$ increasingly emerging as key components of this cascade. In goats, we have previously studied the effects of *E. coli* endotoxins, r.BoTNF-$_\alpha$, r.HuTNF-$_\alpha$, r.HuIFN-$_{\alpha 2a}$ as well as some interferon-inducers.[20–25,30] *E. coli* endotoxin is a potent inducer of TNF-$_\alpha$, IL-$_1$ and IFN.[1,2,15,19,29] Both TNF-$_\alpha$ and IL-$_{1\beta}$ are proximal mediators in the cytokine cascade, appearing in the circulation of several species as brief, early peaks after infusion of bacteria or endotoxin. This transient appearance is sufficient to induce a cascade of secondary cytokines, including IL-$_6$, platelet activating factor and transforming growth factor beta.[2,29] The regulation of cytokines is complex and poorly understood. To improve our understanding of the biology and pathophysiology of primary and secondary cytokines, the usage of specific neutralizing antibodies is therefore of special interest.

Once released into the circulation, pyrogenic cytokines travel from peripheral sites of infection to the brain, where they act on structures in the thermoregulatory centre of the hypothalamus to initiate fever. TNF-$_\alpha$ and IFN-$_\alpha$ induce fever via the same mechanism as that shown for IL-$_1$: synthesis of brain prostaglandin-$E_2$.[31,32]

In the present study, the rate of rise and time of peak elevation in fever induced by r.BoIFN-$_\gamma$ differed from these parameters in r.BoIL-$_{1\beta}$ (Figure 1) or r.BoTNF-$_\alpha$ fever[30] and were similar to the values obtained after injection of low doses of endotoxin. No data are available on whether IFN-$_\gamma$ induces the production of IL-$_1$ or TNF-$_\alpha$ *in vivo*. *In vitro*, IFN-$_\gamma$ stimulated prostaglandin-$E_2$ production by mouse Kupffer cells.[33] However, IFN-$_\gamma$ did not directly stimulate prostaglandin-$E_2$ synthesis from rabbit hypothalamic tissue *in vitro*.[19]

The rapid rise in body temperature that occurred in dwarf goats after i.v. injection of r.BoIL-$_{1\beta}$ is indistinguishable from that produced by r.BoTNF-$_\alpha$.[30] Similar results have been reported for rabbits.[19] Injected intravenously into rabbits and dwarf goats, r.IFN-$_\alpha$ induced monophasic fevers that peaked later (after 80–90 min) than those after r.IL-$_1$ or r.TNF-$_\alpha$.[19,20] The delayed onset of fever induced by IFN-$_\gamma$, which was also observed in rabbits,[19,34] and its rather persistent character (at 8 h after injection, 1.34 ± 0.16°C), suggest that fever associated with IFN-$_\gamma$ may be mediated through the induction of other endogenous pyrogens.

Although high doses of r.HuIL-$_2$ (120 $\mu g \cdot kg^{-1}$) caused fever in rabbits, low doses (3 $\mu g \cdot kg^{-1}$ i.v.) were nonpyrogenic.[18] In sheep, high doses of r.IL-$_2$ (100 $\mu g \cdot kg^{-1}$ i.v.) induced side effects and increased plasma levels of prostaglandin-$F_{2\alpha}$, 6-keto-PGF-$_{1\alpha}$, and thromboxane-$B_2$.[35] These effects were probably due to inducible pyrogens.[18] In the present study, low doses of r.BoIL$_{-2}$ (1 $\mu g \cdot kg^{-1}$ i.v.) were nonpyrogenic in dwarf goats (Figure 1).

Animals infected with Gram-negative bacteria characteristically exhibit in addition to fever, a lowering of plasma Zn and Fe concentrations.[6,36–38] Similar effects can be induced by i.v. injection or intramammary administration of endotoxin.[1,3,24,25] Endotoxins and infectious agents activate polymorph neutrophils to release antibacterial factors including lactoferrin,[39–41] and macrophages to produce cytokines like TNF$_{-\alpha}$ and IL$_{-1}$.[19,29] Once released into the circulation, these cytokines travel from peripheral sites of infection to the brain, liver, spleen and other tissues. Within the brain they induce fever, whereas in the liver they stimulate *de novo* synthesis of serum transferrin,[42] and metallothionein,[43,44] and increase zinc[43] and iron uptake by the hepatocyte.[45] In laboratory animal species, cytokines such as TNF$_{-\alpha}$, IL$_{-1}$ and IFN$_{-\gamma}$ induce hypoferraemia and hypozincaemia with lowest values occurring after 8 h.[2,6,29,34,40,42,46,47]

In concert, in the present study plasma Zn and Fe levels were markedly decreased in goats after i.v. injection of r.BoIL$_{-1\beta}$ (Figure 1). Interestingly, r.BoIFN$_{-\gamma}$ induced only a slight reduction in plasma concentration of these trace metals, while the temperature responses were more pronounced than those after r.BoIL$_{-1\beta}$. Furthermore, neither crude caprine endogenous pyrogen[3] nor i.v. r.HuIFN$_{-\alpha 2a}$[20] induced changes in plasma Zn and Fe concentrations in dwarf goats.

In contrast to i.v. administration, i.m. injection of r.HuIFN$_{-\alpha 2a}$ (at a 10 times greater dose) caused hypoferraemia and hypozincaemia.[20] It is likely that local irritation at the injection site and the much higher dose of r.HuIFN$_{-\alpha 2a}$ used in that study, might have caused the synthesis and release of other pyrogenic cytokines. The decreases in plasma trace metal concentrations induced by r.BoIL$_{-1\beta}$ were essentially the same as those described previously for r.BoTNF$_{-\alpha}$ and *E. coli* endotoxin.[30] However, the time of onset of these changes after the injection of *E. coli* endotoxin was markedly longer than after r.BoIL$_{-1\beta}$ or r.BoTNF$_{-\alpha}$ administration. Taken together, these results suggest that IL$_{-1\beta}$ and TNF$_{-\alpha}$ rather than IFN$_{-\alpha}$ and IFN$_{-\gamma}$ are likely to be the cytokines responsible for hypoferraemia and hypozincaemia. Based on these observations, it seems unlikely that fever induced by r.BoIFN$_{-\gamma}$ is due to the synthesis and release of IL$_{-1}$ and TNF$_{-\alpha}$.

In the present study, the haematologic changes were cell-type and cytokine specific. Transient lymphopenia was observed after administration of all three cytokines, reaching a nadir 3.5 to 8 h after i.v. injection. Neutrophilia developed after i.v. injection of r.BoIL$_{-2}$ and r.BoIFN$_{-\gamma}$; similar results have been reported for man.[48] The haematological changes induced by r.BoIL$_{-1\beta}$ were characterized by lymphopenia and initial neutropenia that was followed by neutrophilia (Table 1). In a previous study, similar results have been obtained with *E. coli* endotoxin.[32] In comparison with r.BoTNF$_{-\alpha}$,[32] r.BoIL$_{-1\beta}$ induced less lymphopenia, a shorter initial neutropenia and a greater neutrophilia in dwarf goats. Moreover, only r.BoIL$_{-1\beta}$ induced a significant increase in the number of circulating immature neutrophils. Since endotoxin causes mononuclear cells to release both TNF$_{-\alpha}$ and IL$_{-1}$, these cytokines may be presumed to be important mediators of endotoxin-induced haematological changes. It seems likely that the endotoxin-induced neutropenia is mainly caused by TNF$_{-\alpha}$, whereas the endotoxin-induced neutrophilia and associated increases in immature neutrophils are due to the combined effects of IL$_{-1}$ and TNF$_{-\alpha}$.

In summary, we conclude that r.BoIL$_{-1\beta}$ induces many of the biological changes that characterize the acute phase response to endotoxins[21,22,24,25,32] or Gram-negative bacterial infections.[36–38] In dwarf goats, the effects induced by r.BoIL$_{-1\beta}$, as described above (biphasic fever, reduction in plasma Zn and Fe concentrations and haematologic changes), together with the clinical effects reported elsewhere (changes in feeding behaviour, forestomach motility and heart rate, see Ref. 23), resemble those observed after i.v. injection of r.BoTNF$_{-\alpha}$.[32] However, the time of onset of the effects after injection of these cytokines was significantly shorter than after endotoxin. These findings are in accordance to the well-documented role of IL$_{-1}$ and TNF$_{-\alpha}$ as pivotal mediators of endotoxin toxicity. The biological activities of r.BoIL$_{-1\beta}$ in dwarf goats may indicate that r.BoIL$_{-1\beta}$ has some homology with caprine IL$_{-1}$. Furthermore, we conclude that r.BoIFN$_{-\gamma}$ induces effects which resemble those observed after i.v. injection of interferon inducers and IFN$_{-\alpha}$.[20–22] These biological changes are characteristic for the acute phase response to infection. However, latency time, intensity and duration of these effects are markedly different from those observed after IL$_{-1\beta}$ and TNF$_{-\alpha}$. The delayed onset of IFN-induced effects suggests that they may be mediated through the induction of other mediators of inflammation. Further investigations remain to be completed before the role of each cytokine, or combinations of cytokines, can be established in a comprehensive explanation for the pathogenesis of febrile conditions to viral, protozoal and bacterial infections.

### References

1. Dinarello CA. Interleukin-1. *Rev Infect Dis* 1984; **6**: 51–95.
2. Dinarello CA. Interleukin-1 and its biologically related cytokines. *Adv Immunol* 1989; **44**: 153–205.

3. Van Miert ASJPAM, Van Duin CTM, Verheijden JHM, Schotman AJH, Nieuwenhuis J. Fever and changes in plasma zinc and iron concentrations in the goat: the role of leukocytic pyrogen. *J Comp Path* 1984; **94**: 543–557.
4. Van Miert ASJPAM. Acute phase response and non cellular defence mechanisms. In: Burvenich C, Vandeputte-van Messom G, Hill AW, eds. *New Insights Into The Pathogenesis of Mastitis.* Ghent: Vlaams Diergeneeskundig Tijdschrift, 1991; 69–91.
5. Kluger MJ. The febrile response. In: *Stress Proteins In Biology And Medicine.* Cold Spring Harbour Lab. Press, 1990; 61–78.
6. Weinberg ED. Cellular iron metabolism in health and disease. *Drug Metab Rev* 1990; **22**: 531–579.
7. Beutler B, Milssark IW, Cerami A. Cachectin/tumour necrosis factor: production, distribution, and metabolic fate *in vivo*. *J Immunol* 1985; **135**: 3972–3977.
8. Adams JL, Semrad SD, Czuprynski CJ. Administration of bacterial lipopolysaccharide elicits circulating tumour necrosis factor-alpha in neonatal calves. *J Clin Microbiol* 1990; **28**: 998–1001.
9. Girardin E, Brau GE, Dayer JM, *et al.* Tumour necrosis factor and interleukin-1 in the serum of children with severe infectious purpura. *N Engl J Med* 1988; **319**: 397–400.
10. Henricson BE, Benjamin WR, Vogel SN. Differential cytokine induction by doses of lipopolysaccharide and monophosphoryl lipid A that result in equivalent early endotoxin tolerance. *Infect Immun* 1990; **58**: 2429–2437.
11. Kumazawa Y, Freudenberg MA, Hausmann C, Meding-Slade S, Langhorne J, Galanos C. Formation of interferon-gamma and tumour necrosis factor in mice during *Salmonella typhimurium* infection. *Pathobiol* 1991; **59**: 194–196.
12. Minamishima I, Ohga S, Ishii E, *et al.* Aseptic meningitis in children: correlation between fever and interferon-gamma level. *Eur J Pediatr* 1991; **150**: 722–725.
13. Abbot RJ, Bolderson I, Gruer PJK. $IFN_{-\gamma}$ and $IFN_{-\alpha}$ in CSF in viral meningitis. *Lancet* 1985; **11**: 456–457.
14. McCracken GH, Mustafa MM, Ramilo O, Olsen KD, Risser RC. Cerebrospinal fluid interleukin-1 beta and tumour necrosis factor concentrations and outcome from neonatal gram-negative enteric bacillary meningitis. *Pediatr Infect Dis* 1989; **8**: 155–159.
15. Kluger MJ. Fever: role of pyrogens and cryogens. *Physiol Rev* 1991; **71**: 93–127.
16. Lauria F, Zinzani PL, Rondelli D, Raspadori D, Benfenati D, Tura S. Continuous infusion of interleukin-2 in two relapsed high grade non-Hodgkin lymphoma patients: effectiveness and tolerability. *Eur J Cancer* 1991; **27**: 521–522.
17. Rosenberg SA, Lotze MT, Muul LM, *et al.* A progress report on the treatment of 157 patients with advanced cancer using lymphokine-activated killer cells and interleukin-2 or high-dose interleukin-2 alone. *N Engl J Med* 1987; **316**: 889–897.
18. Mier JW, Vachino G, Van der Meer JWM, *et al.* Induction of circulating tumor necrosis factor ($TNF_{\alpha}$) as the mechanism for the febrile response to interleukin-2 (IL-2) in cancer patients. *J Clin Immunol* 1988; **8**: 426–437.
19. Dinarello CA, Cannon JP, Wolff SM. New concepts on the pathogenesis of fever. *Rev Infect Dis* 1988; **10**: 168–189.
20. Van Miert ASJPAM, Van Duin CTM, Wensing T. Fever and changes in plasma zinc and iron concentrations in the goat. The effects of interferon-inducers and recombinant $IFN_{-\alpha2a}$. *J Comp Path* 1990; **103**: 289–300.
21. Van Miert ASJPAM, Van Duin CTM, Koot M. Effects of *E. coli* endotoxin, some interferon-inducers, recombinant $interferon_{-\alpha2a}$ and *Trypanosoma brucei* infection on feed intake in dwarf goats. *J Vet Pharmacol Therap* 1990; **13**: 327–331.
22. Koot M, Van Duin CTM, Wensing T, Van Miert ASJPAM. Comparative observations of fever and associated clinical, haematological and blood biochemical changes after parenteral administration of Poly I: Poly C, $interferon\text{-}alpha_{2a}$ and *Escherichia coli* endotoxin in goats. *Vet Quarterly* 1989; **11**: 41–50.
23. Van Miert ASJPAM, Kaya F, Van Duin CTM. Changes in food intake and forestomach motility of dwarf goats by recombinant-deprived bovine $IL_{-1\beta}$, $IL_{-2}$ and $IFN_{-\gamma}$. Submitted for publication.
24. Van Miert ASJPAM, Van Duin CTM, Verheijden JHM, Schotman AJH. Endotoxin-induced fever and associated haematological and blood biochemical changes in the goat. The effect of repeated administration and the influence of flurbiprofen. *Res Vet Sci* 1982; **33**: 248–255.
25. Van Miert ASJPAM, Van Duin CTM, Verheijden JHM, Schotman AJH. Staphylococcal enterotoxin-B and *Escherichia coli* endotoxin: comparative observations in goats on fever and associated clinical haematologic and blood biochemical changes after i.v. and intramammary administration. *Am J Vet Res* 1983; **44**: 955–963.
26. Murakami N, Sakata Y, Watanabe T. Central action sites of $interleukin_{-1\beta}$ for inducing fever in rabbits. *J Physiol (London)* 1990; **428**: 299–312.
27. Roth JA, Frank DE. Recombinant bovine $interferon_{-\gamma}$ as an immunomodulator in dexamethasone-treated and nontreated cattle. *J Interfer Res* 1989; **9**: 143–151.
28. Dinarello CA. The pathophysiology of the pro-inflammatory cytokines. *Biotherapy* 1990; **2**: 189–191.
29. Durum SK, Oppenheim JJ. Macrophage-derived mediators: $interleukin_{-1}$, tumour necrosis factor, $interleukin_{-6}$, interferon, and related cytokines. In: Paul WE ed. *Fundamental Immunology.* 2nd edition. New York: Raven Press, 1989: 639–661.
30. Van Miert ASJPAM, Van Duin CTM, Wensing T. Fever and acute phase response induced in dwarf goats by endotoxin and bovine and human recombinant tumor necrosis factor-alpha. *J Vet Pharmacol Therap* 1992; in press.
31. Coceani F, Lees J, Bishail I. Further evidence implicating prostaglandin-E2 in the genesis of pyrogen fever. *Am J Physiol* 1988; **245**: R463–R469.
32. Nakamura H, Seto Y, Motoyoshi S, Kadokawa T, Sunahara N. Recombinant human tumour necrosis factor causes long-lasting and prostaglandin-mediated fever, with little tolerance in rabbits. *J Pharmacol Exp Ther* 1988; **245**: 336–341.
33. Kawada N, Mizoguchi Y, Shin T, *et al.* $Interferon_{-\gamma}$ stimulates prostaglandin-E2 production by mouse Kupffer cells. *Prostagland Leuk Essent Fatty Acids* 1990; **40**: 275–279.
34. Morimoto A, Murakami N, Takada M, Teshiroji S, Watanaba T. Fever and acute phase response induced in rabbits by human recombinant $interferon_{-\gamma}$. *J Physiol (London)* 1987; **391**: 209–218.
35. O'Neill CA, Gunther RA, Jesmok GJ, Giri SN. Effects of recombinant human $interleukin_{-2}$ and excipient infusion on plasma levels of prostaglandins and thromboxane B2 in sheep. *Lymphokine Cytokine Res* 1991; **10**: 207–212.
36. Groothuis DG, Van Miert ASJPAM, Schotman AJH. Zinc concentration in plasma during experimental *Salmonella dublin* infection and endotoxin-induced fever in calves. *Vet Rec* 1981; **109**: 176–177.
37. Lohuis JACM, Schukken YH, Verheijden JHM, Brand A, Van Miert ASJPAM. Effect of severity of systemic signs during the acute phase of experimentally induced *Escherichia coli* mastitis on milk production losses. *J Dairy Sci* 1990; **73**: 333–341.
38. Mevius DJ, Breukink HJ, Van Miert ASJPAM, Kessels BGF, Jobse AS, Smit JAH. Effects of experimentally induced *Pasteurella haemolytica* infection in dairy calves on the pharmacokinetics of flumequine. *J Vet Pharmacol Therap* 1991; **14**: 174–184.
39. Gutteberg TJ, Røkke O, Andersen O, Jørgensen T. Lactoferrin as an indicator of septicemia and endotoxemia in pigs. *Scand J Infect Dis* 1988; **20**: 659–666.
40. Goldblum SE, Cohen DA, Jay M, McClain J. $Interleukin_{-1}$-induced depression of iron and lactoferrin. *Am J Physiol* 1987; **252**: E27–E32.
41. Koivuranta-Vara P, Banda D, Goldstein IM. Bacterial-lipopolysaccharide-induced release of lactoferrin from human polymorph leukocytes: role of monocyte-derived tumour necrosis factor alpha. *Infect Immun* 1987; **55**: 2956–2961.
42. Beaumier DL, Caldwell MS, Holbein BE. Inflammation triggers hypoferremia and *de novo* synthesis of serum transferrin and ceruloplasmin in mice. *Infect Immun* 1984; **46**: 489–494.
43. Cousins RJ. Absorption, transport, and hepatic metabolism of copper and zinc: special reference to metallothionein and ceruloplasmin. *Physiol Rev* 1985; **65**: 238–309.
44. Liu J, Liu YP, Sendelbach LE, Klaassen CD. Endotoxin induction of hepatic metallothionein is mediated through cytokines. *Toxicol Appl Pharmacol* 1991; **109**: 235–240.
45. Potter BJ, Blades B, McHugh TA, *et al.* Effects of endotoxin on iron uptake by the hepatocyte. *Am J Physiol* 1989; **257**: G524–G531.
46. Wada M, Morimoto A, Watanabe T, Sakata Y, Marakami N. Effects of physical training on febrile and acute-phase responses induced in rats by bacterial endotoxin or $interleukin_{-1}$. *J Physiol (London)* 1990; **430**: 595–603.
47. Fischer E, Marano MA, Barber AE, *et al.* Comparison between effects of $interleukin_{-1\alpha}$ administration and sublethal endotoxemia in primates. *Am J Physiol* 1991; **261**: R442–R452.
48. Aulitzky WE, Tilg H, Vogel W, *et al.* Acute hematologic effects of interferon alpha, interferon gamma, tumour necrosis factor alpha and $interleukin_{-2}$. *Ann Hematol* 1991; **62**: 25–31.

ACKNOWLEDGEMENTS. We are grateful to Ciba-Geigy Ltd, Basle, Switzerland (Dr R. F. Steiger) and American Cyanamid Company, Princeton, NJ, USA (Dr T. J. Williams) for their generous gifts of $r.BoIFN_{-\gamma}$, $r.BoIL_{-1\beta}$ and $r.BoIL_{-2}$, respectively. The technical assistance of Mrs E. Simcox and Mr J. M. Eyndhoven in preparing the manuscript is greatly appreciated.

**Received 28 February 1992; accepted with revision 7 April 1992**
